# Diagnostic value of next-generation sequencing to detect periprosthetic joint infection

**DOI:** 10.1186/s12891-021-04116-9

**Published:** 2021-03-06

**Authors:** Han Yin, Duliang Xu, Dawei Wang

**Affiliations:** Department of Orthopaedics, Liaocheng People’s Hospital and Liaocheng Clinical School of Taishan Medical University, No. 67, Dongchang Road, Liaocheng, Shandong China

**Keywords:** Arthroplasty, Serological biomarkers, Bacterial culture, Next-generation sequencing, Joint replacement, Periprosthetic joint infection

## Abstract

**Background:**

We herein compared the diagnostic value of next-generation sequencing (NGS), bacterial culture, and serological biomarkers to detect periprosthetic joint infection (PJI) after joint replacement.

**Methods:**

According to the diagnostic criteria of the Musculoskeletal Infection Society, 35 patients who underwent joint revision surgery were divided into infection (15 cases) and non-infection (20 cases) groups, and were routinely examined preoperatively for erythrocyte sedimentation rate (ESR), C-reactive protein (CRP), procalcitonin (PCT), interleukin-6 (IL-6), and D-dimer levels. All patients underwent arthrocentesis preoperatively. Synovial fluid was used for white blood cell count, white blood cell classification, bacterial culture, and NGS. Furthermore, we calculated the area under the curve (AUC) of the receiver operating characteristic curve (ROC) for ESR, CRP, PCT, IL-6, and D-dimer. Data were assessed by comparing diagnostic accuracy, sensitivity, and specificity.

**Results:**

Fourteen patients showed positive results by NGS and seven showed positive bacterial culture results in the infection group; further, 18 showed negative results by NGS in the non-infection group. The AUC of ESR, D-dimer, CRP, IL-6, and PCT was 0.667, 0.572, 0.827, 0.767, and 0.808, respectively. The accuracy of NGS, bacterial culture, CRP, IL-6, and PCT was 0.91, 0.74, 0.77, 0.74, and 0.83, respectively. When comparing NGS with CRP, IL-6, PCT, and bacterial culture, differences in overall test results and those in sensitivity were statistically significant, and compared with CRP, differences in specificity were also statistically significant. In comparison with IL-6, PCT, and bacterial culture, the specificity of NGS was statistically insignificant.

**Conclusions:**

Our results indicated that NGS had higher accuracy and sensitivity than the bacterial culture method and commonly used serological biomarkers for diagnosing PJI.

## Background

Periprosthetic joint infection (PJI) is the most common cause of total knee arthroplasty failure (20.4%) [1] and one of the main reasons for revision after total knee arthroplasty (12.8%) [2]. Such a revision usually demands multiple surgical interventions and has a higher incidence of complications. The mortality rate of patients with PJI undergoing arthroplasty is five times that of patients undergoing aseptic arthroplasty [3, 4].

The main difficulty associated with PJI is prompt and accurate diagnosis, along with the detection of pathogens. Due to complicated reasons such as the existence of biofilms on the surface of artificial joints, inconsistent and nonoptimal processes for sampling pathogenic bacteria, small number of bacteria, and complex symbiotic relationship involving bacteria, pathogens cannot be easily detected in approximately 50% patients with PJI. Compared with culture-positive cases, the rate of infection recurrence is 4–5 times higher in culture-negative cases [5].

Several biomarkers have been studied in recent years to improve the accuracy of diagnosing PJI, such as C-reactive protein (CRP) [6], α-defensin [7], leukocyte esterase [8], interleukin-6 (IL-6) [9], vascular endothelial growth factor [10], and granulocyte colony-stimulating factor [11], but the overall sensitivity (77–97%) and specificity (86–96%) remain inadequate [12]. PCR was once considered a reliable method for diagnosing PJI [13]. Studies on the use of PCR to diagnose PJI continue to be extensively conducted, but the sensitivity of the method has been observed to widely vary. A meta-analysis reported that the overall sensitivity of PCR was only 76%, which is comparable to that of traditional culture [14].

Next-generation sequencing (NGS) is an emerging microbial diagnostic method that can detect all nucleic acids present in a specimen at once, including those from the host. For example, NGS has been reported to successfully detect pathogenic bacteria in samples from patients with nervous system infections [15] and sepsis [16]. Further, it has been used for identifying pathogenic organisms in medical systems and is associated with a good clinical value. We conducted this study because at present, very few studies have explored the use of NGS for diagnosing PJI [5, 17], and thus, its diagnostic value in case of PJI remains unclear.

## Methods

### Patients

We selected 35 patients who underwent joint revision surgery (involving hip and knee joints) at our department from July 2017 to December 2019. Patients diagnosed with PJI and aseptic loosening, as defined by Musculoskeletal Infection Society (MSIS) criteria [18], were included. Individuals were excluded if no fluid could be aspirated prior to injecting sterile normal saline and if infected lesions were present in other parts of their body. The patients were divided into two groups: infection and non-infection.

### Preoperative specimens and detection indicators

Preoperative CRP, ESR, procalcitonin (PCT), IL-6, and D-dimer levels were routinely examined.

The CRP and ESR were tested with C-Reactive Protein Rapid Test Kit (Easydiagnosis Biomedicine co. Ltd., China) and Erythrocyte Sedimentation Rate Test Kit (Jianglaibio, China) respectively. PCT was tested with PCT ELISA kit (MSK co. ltd, China). IL-6 and D-dimer were tested with IL-6 ELISA kit (MSK co. ltd, China) and D-dimer ELISA kit (MSK co. ltd, China). Synovial fluid was used for white blood cell count, white blood cell classification. Joint fluid was respectively injected into aerobic, anaerobic and blood culture bottles and cultivated for 14d.

The receiver operating characteristic curve was used to analyze results pertaining to CRP, ESR, IL-6, PCT, and D-dimer, to evaluate diagnostic value for PJI, and to determine the optimal cut-off value according to the Youden index (sensitivity + specificity − 1). The accuracy, sensitivity, specificity, positive and negative predictive values, and positive and negative likelihood ratio of each detection method were simultaneously calculated.

### NGS

For NGS, 0.6 mL specimen was transferred to a 2 mL microcentrifuge tube containing 0.3 mL of 0.5 mm glass beads. The tube was then attached to a horizontal platform on a vortex mixer and vigorously agitated at 2800–3200 rpm for 20 min. DNA was extracted using the TIANamp Micro DNA Kit (DP316, TIANGEN BIOTECH), according to manufacturer instructions. The extracted DNA was quantified by Qubit 2.0 (Invitrogen, USA) and up to 200 ng was used for library construction.

DNA library was constructed by DNA fragmentation, end repair, and PCR amplification. Quality was assessed using Agilent 2100 (Agilent Technologies) and Qubit 2.0 (Invitrogen). Double-stranded DNA was then converted into single-stranded circular DNA via degradation and cyclization. DNA nanospheres were subsequently generated by rolling circle amplification. Qualified DNA nanospheres were loaded onto a chip and then subjected to 20 M 50-bp single-end sequencing on the BGISEQ-50 platform (BGI Genomics Co., Ltd.).

High quality sequencing data was obtained by removing low quality, short reads (length < 35 bp), followed by computational subtraction of human host sequences mapped to the human reference genome (hg19) using the Burrows–Wheeler alignment tool. The remaining data by removal of low-complexity reads were classified by simultaneously aligning to four Microbial Genome Databases, comprising viruses, bacteria, fungi, and parasites. The databases were downloaded from NCBI (ftp://ftp.ncbi.nlm.nih.gov/genomes/) and contained 1798 whole genome sequences of viral taxa, 6350 bacterial genomes or scaffolds, 1064 fungi related to human infection, and 234 parasites associated with human diseases.

In this study, we used the method of Street et al., [19] to define the positive NGS results. When the RAG (Relative abundance in genus⁃ level) of the bacteria is calculated to be 10% ~ 35%, the pathogen with the highest Youden index (sensitivity + specificity-1) for the diagnosis of PJI and the least missed culture-positive pathogen is regarded as the pathogen.

### Statistical methods

SPSS 24 (IBM, Armonk, NY, United States) was used for statistical analysis. We used descriptive statistics to display baseline characteristics. Two groups of continuous variables were compared by t-test or nonparametric test (Mann–Whitney U test), and categorical variables were assessed using chi-square test. McNemar test was used to compare the two methods. *P* < 0.05 indicated statistical significance.

## Results

**Fig. 1:**
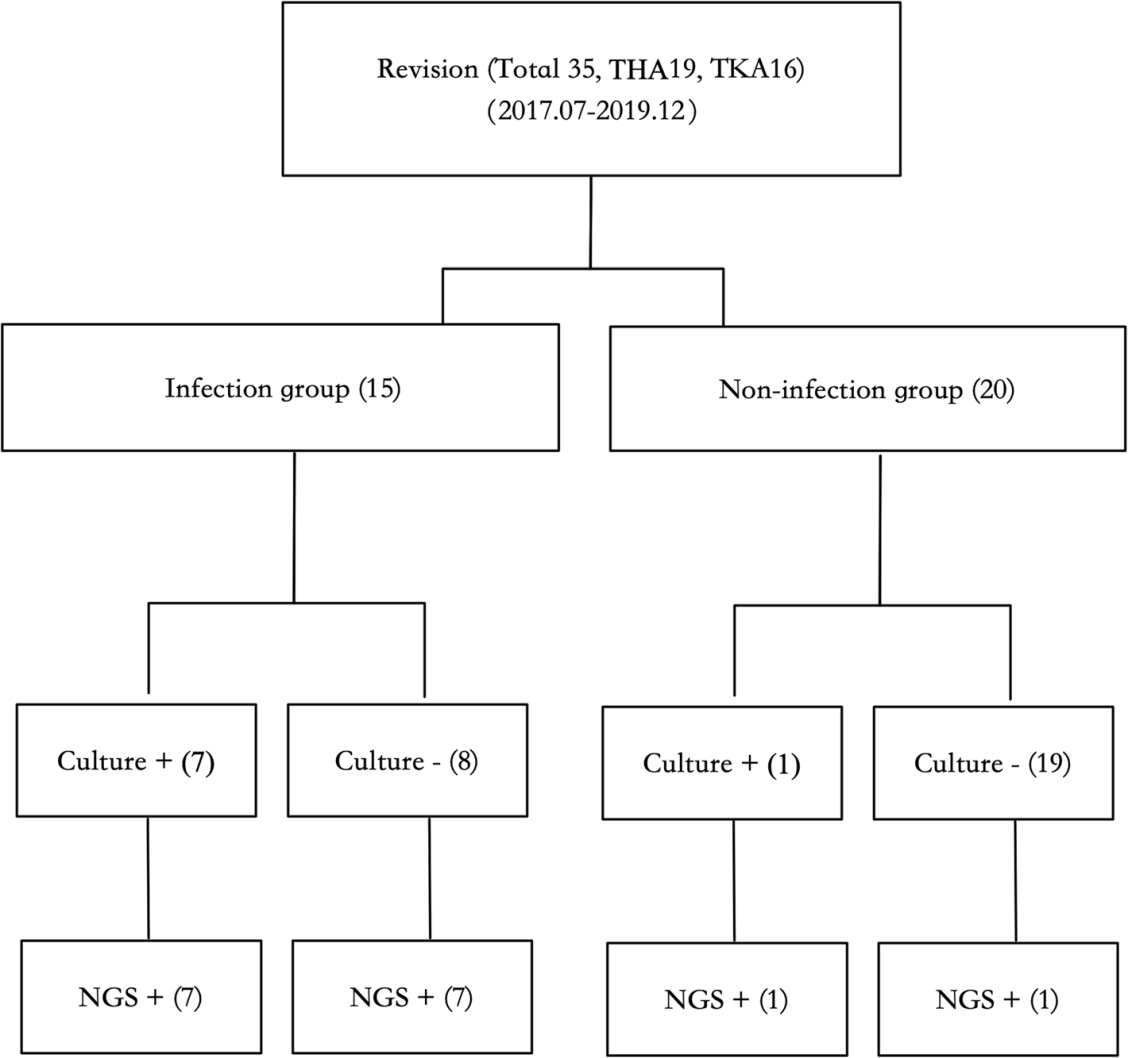
Patient selection flowchart. THA Total Hip Arthroplasty, TKA Total Knee Arthroplasty, + Positive, − Negative

Based on the MSIS criteria, the infection and non-infection groups included 15 and 20 patients, respectively (Fig. 1). There were no significant differences in age, gender, body mass index, and affected joints between the groups. There were seven cases (46.7%) of hip joints and eight (53.3%) of knee joints in the infection group, and there were 12 cases (60.0%) of hip joints and eight (40.0%) of knee joints in the non-infection group. No statistically significant difference was noted in the composition ratio between the groups (*P* > 0.05; Table 1).

**Table 1:** Baseline patient characteristics

	Total	Infection group	Non-infection group	*P* value
Age (years)	67.8 ± 7.4	66.4 ± 7.6	68.8 ± 7.2	0.348
	67 (62–74)	67 (63–69.5)	68.5 (62–74)	0.458^a^
Sex				0.728^b^
Men	21 (60)	10 (66.7)	11 (55)	
Women	14 (40)	5 (33.3)	9 (45)	
BMI	27.2 ± 2.3	26.9 ± 2.4	27.4 ± 2.1	0.550
Affected joint				0.506^b^
Hip	19 (54.3)	7 (46.7)	12 (60)	
Knee	16 (45.7)	8 (53.3)	8 (40)	

^a^Mann-Whitney U test, ^b^Fisher exact test, *BMI* Body Mass Index

In the infection group, 14/15 (93.3%) patients showed positive results via NGS and 7/15 (46.7%) had positive bacterial culture results, whereas in the non-infection group, 18/20 (90.0%) patients showed negative results via NGS and 1/20 (5.0%) had bacterial culture results. The mNGS positive results of infection group included 6 cases of *Staphylococcus aureus*, 2 cases of *Pseudomonas aeruginosa*, 2 cases of *Staphylococcus epidermidis*, 2 cases of *Streptococcus pyogenes*, 1 case of Staphylococcus lugdunensis, and 1 case of *Clostridium perfringens*. The culture positive results of the infection group included 3 cases of *Staphylococcus aureus*, 2 cases of *Streptococcus pyogenes*, and 1 case of *Clostridium perfringens*,1 case of Staphylococcus. The mNGS positive results of non-infection group included 1 case of *Candida glabrata* and 1 case of *Clostridium perfringens*. The culture positive results of the infection group included 1 case of *Candida glabrata* (Table 2). NGS and bacterial culture results showed significant differences between the infection (*P* < 0.01) and non-infection groups (*P* = 0.011).

**Table 2:** mNGS and culture results of infection group and non-infection group

Number	Group	mNGS	CovRate	Depth	SD SMRN	Culture
1	Infection	Staphylococcus lugdunensis	0.2132	1	26	Staphylococcus
2	Infection	*Staphylococcus aureus*	10.33	1.07	6941	Staphylococcus aureus
3	Infection	*Clostridium perfringens*	2.68	1.02	1172	Clostridium perfringens
4	Infection	Staphylococcus aureus	0.4439	1	183	Staphylococcus aureus
5	Infection	*Streptococcus pyogenes*	0.8676	1	864	Streptococcus pyogenes
6	Infection	Streptococcus pyogenes	0.0646	1	78	Streptococcus pyogenes
7	Infection	Staphylococcus aureus	0.764	1	256	Staphylococcus aureus
8	Infection	*Staphylococcus epidermidis*	0.058	1	56	–
9	Infection	Staphylococcus aureus	9.34	1	5624	–
10	Infection	Staphylococcus aureus	0.672	1	423	–
11	Infection	*Pseudomonas aeruginosa*	0.146	1	314	–
12	Infection	Pseudomonas aeruginosa	0.084	1	22	–
13	Infection	Staphylococcus epidermidis	8.4	1	2385	–
14	Infection	Staphylococcus aureus	0.106	1	24	
15	Non-infection	*Candida glabrata*	2.033	1	4221	Candida glabrata
16	Non-infection	Clostridium perfringens	0.092	1	44	–

*mNGS* Metagenomics next generation sequencing, *CovRate* Coverage Rate, *SD* SMRN Standardized Stringently mapped reads number in species level

**Fig. 2:**
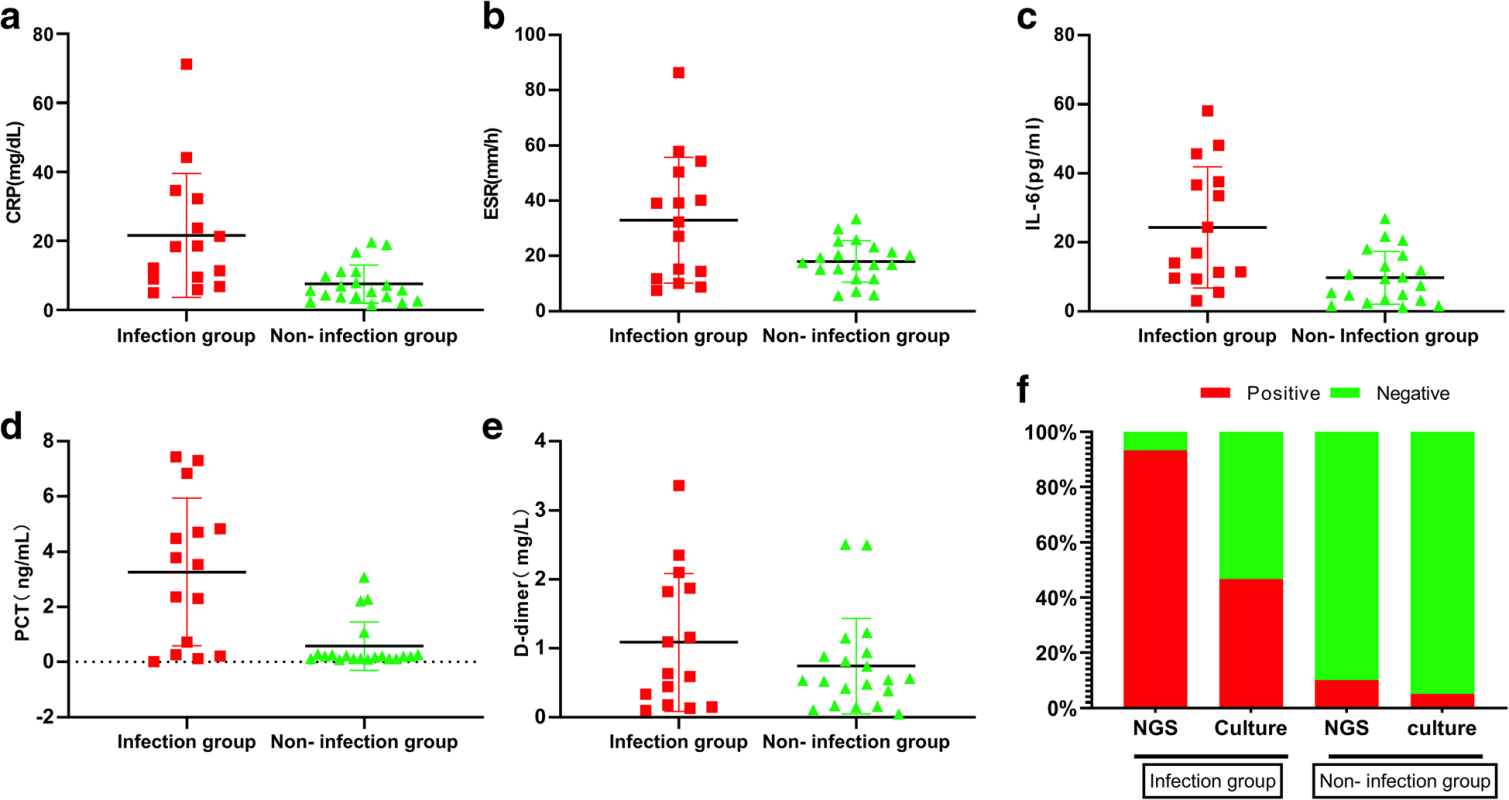
Comparison of different serum biomarkers levels between infection group and non-infection group. **a** The CRP value of the infecton group was significantly higher than that of the non-infection group (*P* = 0.001), **b** There was not significantly different of ESR value between infection group and non-infection group (*P* = 0.099), **c** The IL-6 value of the infecton group was significantly higher than that of the non-infection group (*P* = 0.007), **d** The PCT value of the infecton group was significantly higher than that of the non-infection group (*P* = 0.001), **e** There was not significantly different of D-dimer level between infection group and non-infection group (*P* = 0.479), **f** 14/15 (93.3%) patients showed positive results via NGS and 7/15 (46.7%) had positive bacterial culture results in the infection group, whereas in the non-infection group, 18/20 (90.0%) patients showed negative results via NGS and 1/20 (5.0%) had bacterial culture results

CRP levels were significantly higher in the infection group [18.3 (8.9–32.2) mg/dL] than in the non-infection group [5.7 (3.7–10.8) mg/dL)] (*P* = 0.001). The difference in ESR levels was not statistically significant between the groups [32.3 (11.7–50.5) mm/h vs. 17.2 (12.6–22.8) mm/h, *P* = 0.099]. The levels of IL-6 and PCT were 16.8 (9.5–37.6) pg/mL and 3.5 (0.3–4.8) ng/mL in the infection group, respectively, which were significantly higher than those in the non-infection group [8.4 (3.3–15.5) pg/mL and 0.2 (0.1–0.3) ng/mL, *P* = 0.007 and P = 0.001, respectively]. There was no statistically significant differences in D-dimer levels between the groups [0.6 (0.2–1.9) mg/L vs. 0.5 (0.2–0.9) mg/L, *P* = 0.479] (Table 3, Fig. 2).

**Table 3:** NGS, bacterial culture and serum biomarker test results

	Infection group	Non-infection group	*P* value
CRP (mg/dL)	18.3 (8.9–32.2)	5.7 (3.7–10.8)	0.001^a^
ESR (mm/h)	32.3 (11.7–50.5)	17.2 (12.6–22.8)	0.099^a^
IL-6(pg/ml)	16.8 (9.5–37.6)	8.4 (3.3–15.5)	0.007^a^
PCT (ng/mL)	3.5 (0.3–4.8)	0.2 (0.1–0.3)	0.001^a^
D-Dimer (mg/L)	0.6 (0.2–1.9)	0.5 (0.2–0.9)	0.479^a^
NGS			< 0.001^b^
+	14 (93.3)	2 (10)	
-	1 (6.7)	18 (90)	
Bacterial culture			0.011^b^
+	7 (46.7)	1 (5)	
_	8 (53.3)	19 (95)	

^a^Mann-Whitney U test, ^b^Fisher exact test, + positive, - negative

**Fig. 3:**
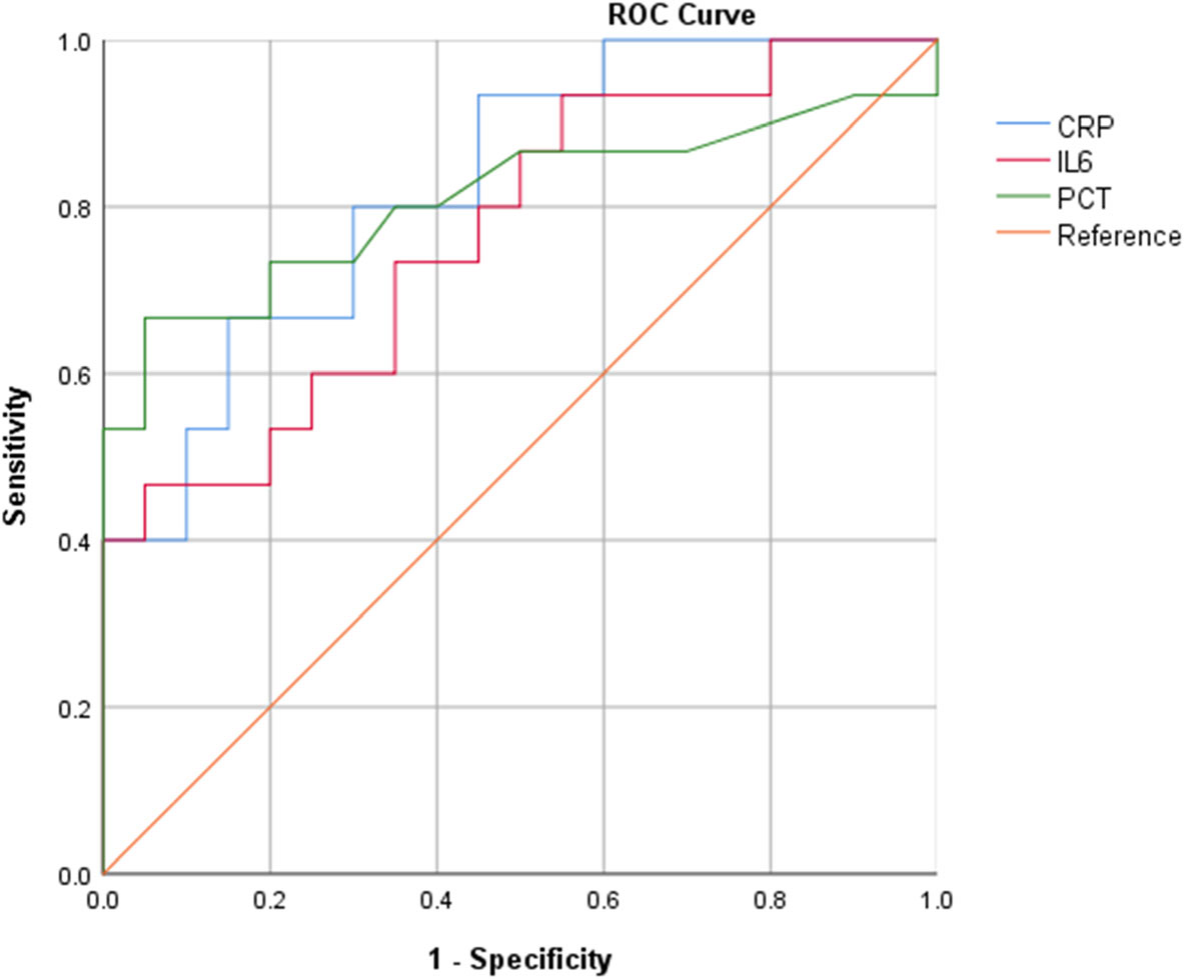
The receiver operating characteristic curve (ROC) for CRP, PCT, IL-6. The AUC of CRP, IL-6, and PCT was 0.827, 0.767, and 0.808, respectively (*P* < 0.05)

The area under the curve (AUC) of ESR and D-dimer was 0.667 and 0.572, respectively (*P* > 0.05). The AUC of CRP, IL-6, and PCT was 0.827, 0.767, and 0.808, respectively (*P* < 0.05). The corresponding clinical diagnostic cut-off values were 11.2 mg/dL for CRP, 23.05 pg/mL for IL-6, and 2.29 ng/mL for PCT (Table 4, Fig. 3). According to this critical point, the sensitivity of CRP, IL-6, and PCT was 0.667, 0.467, and 0.467, respectively, and the specificity was 0.850, 0.95, and 0.95, respectively. The sensitivity of NGS and bacterial culture was 0.933 and 0.467 (*P* < 0.05), respectively, and the specificity was 0.90 and 0.95 (*P* > 0.05), respectively. The accuracy of NGS, bacterial culture, CRP, IL-6, and PCT were 0.91, 0.74, 0.77, 0.74, and 0.83, respectively.

**Table 4:** The AUC and Cutoff value for CRP, ESR, IL-6, PCT and D-dimer

	AUC	SE	P Value	95% CI	Cutoff value
CRP	0.827	0.069	0.001	0.692–0.961	11.20
ESR	0.667	0.107	0.096	0.457–0.876	26.50
IL-6	0.767	0.081	0.008	0.608–0.926	23.05
PCT	0.808	0.085	0.002	0.642–0.774	2.29
D-Dimer	0.572	0.103	0.474	0.369–0.889	1.02

On comparing NGS with CRP, IL-6, PCT, and bacterial culture, the overall test results were statistically significant (*P* < 0.01). Moreover, when comparing IL-6 and PCT with CRP, no significant differences were found in the overall test results (*P* > 0.05), and on comparing NGS with CRP, IL-6, PCT, and bacterial culture, the difference in sensitivity was statistically significant (*P* < 0.05). Compared with CRP, the specificity difference of NGS was statistically significant (P < 0.05), and compared with IL-6, PCT, and bacterial culture, the specificity of NGS was statistically insignificant (*P* > 0.05; Table 5).

**Table 5:** The Diagnostic capabilities of CRP, IL-6, PCT, NGS and bacterial culture

	FP	TP	TN	FN	*P*	ACC	Sen	*P*	Spe	*P*	PPV	NPV	+LR	-LR
CRP	3	10	17	5	< 0.01^*^	0.77	0.67	< 0.05^*^	0.85	< 0.05^*^	0.77	0.77	4.44	0.39
IL-6	1	7	19	8	< 0.01^*^	0.74	0.47	< 0.05^*^	0.95	> 0.05^*^	0.88	0.70	9.33	0.56
PCT	1	10	19	5	< 0.01^*^	0.83	0.67	< 0.05^*^	0.95	> 0.05^*^	0.91	0.79	13.33	0.35
NGS	2	14	18	1	–	0.91	0.93	–	0.90	–	0.88	0.95	9.33	0.07
BC	1	7	19	8	< 0.01^*^	0.74	0.47	< 0.05^*^	0.95	> 0.05^*^	0.88	0.70	9.33	0.56

*FP* False positive, *TP* True positive, *TN* True negative, *FN* False negative, *ACC* Accuracy, *Sen* Sensitivity, *Spe* Specificity, *PPV* Positive predictive value, *NPV* Negative predictive value, *+LR* Positive likelihood ratio, *-LR* Negative likelihood ratio, *compared with NGS, BC bacterial culture

## Discussion

We herein explored the value of NGS for diagnosing PJI. Our results showed that the sensitivity of NGS to diagnose PJI (93.3%) was significantly higher than that of bacterial culture (47%); moreover, NGS showed a higher positive diagnostic rate for culture-negative cases (87.5%). We also compared the diagnostic value of NGS and commonly used serological biomarkers to detect PJI. We found that the accuracy and sensitivity of NGS for diagnosing PJI was significantly higher than those of serological biomarkers.

NGS is widely used to perform metagenetic analyses, and it can simultaneously detect sequences from millions of bases in a single biochemical reaction. In 2005, Margulies et al. performed genome-wide sequencing of *Mycoplasma genitalium* and *Streptococcus pneumoniae*, marking the beginning of the “NGS revolution.” [20] Further, in 2014, Wilson et al. used NGS for the first time to diagnose infectious diseases [16]. NGS could reportedly diagnose clinically uncommon pathogenic bacterial infections and markedly improve the detection of clinically important pathogens, revolutionizing microbial diagnosis. In 2018, Tarabichi et al. reported the use of NGS for diagnosing PJI for the first time. Based on the MSIS criteria, they found that the positive diagnostic rate of NGS was 89.3% for infected cases and 25% for non-infected and culture-negative cases [5]. In the present study, the positive diagnostic rate of NGS was 93.3% for infected cases and 5.3% for non-infected and culture-negative cases. This disparity in results can be attributed to differences in threshold standards. Different NGS platforms, reagents, and databases tend to lead to variances, and NGS detection results also greatly vary. NGS can be not only qualitative but also quantitative, and it can detect the sequence number of bacterial fragments in a specimen. Further studies are warranted to explore the diagnostic significance of the sequence number.

At present, bacterial culture remains the standard method for the definitive diagnosis of PJI; however, the sensitivity of this technique is highly inconsistent (58–95%), which can be attributed to the use of antibiotics before sampling, inconsistency in standardization of sampling and specimen transmission, variances in cultivation time, and presence of unusual bacteria [21, 22]. Berbari et al. found that 53% patients with culture-negative PJI had been treated using antibiotics before sampling. On using large quantities of broad-spectrum antibiotics before sampling, bacterial count reduces or secretions contain antibiotics, consequently affecting bacterial growth and propagation [23]. Moreover, the current bacterial culture time may not be sufficiently long, typically ranging from 3 to 5 days, rather than 2 weeks [24]. In addition, other medical institutions use conventional culture media, which makes the cultivation of microorganisms such as fungi and mycobacteria challenging. Studies have reported that 46% culture-negative PJIs are caused by fungal infections and 43% by mycobacteria [25]. Diagnosing culture-negative PJI is very difficult and may involve a comprehensive evaluation with clinical methods, radiology, serology (inflammatory markers), histopathology, and microbiology. We herein found that the sensitivity of the bacterial culture method to diagnose PJI was 47%, which is lower than the data reported in the literature. This could be because among the eight culture-negative patients with PJI, NGS results indicated that one patient was infected with *Candida albicans*. NGS results also showed that *Staphylococcus aureus* was the most common pathogenic bacteria in prosthetic joint infections. Further, two patients had a history of treatment using broad-spectrum antibiotics. Our findings indicate that bacterial culture and NGS results were generally consistent.

To the best of our knowledge, such an investigation has not been conducted as yet. The ideal method to screen PJI should be highly sensitive so as to minimize false negatives. Serological markers are still the most commonly used method for the clinical diagnosis of PJI. Our results showed that the AUC of ESR and D-dimer was 0.667 and 0.572, respectively, but the results showed no statistical significance. D-dimer, a coagulation-related indicator, has recently been used as a tool for the diagnosis of periprosthetic joint infection (PJI), but its reliability is uncertain. A meta-analysis reported that D-dimer has limited performance for the diagnosis of PJI [26]. The results of our research are the same as reported in the literature. The sensitivity of CRP to diagnose PJI is 67%, which was lower than that reported by the most recent study (85.1%) [27]. The main reason for this is that in this study, the diagnostic cut-off value calculated by the Youden index for CRP was > 11.2 mg/L, which is higher than the cut-off value recommended by the MSIS (> 10 mg/L). A higher cut-off value can lead to a decrease in sensitivity. IL-6 was initially considered to be a highly sensitive and specific marker of PJI [28]. However, because pertinent studies did not consider the confounding effects of previous antibiotic use and related inflammatory conditions on IL-6 and other inflammatory markers, there may have been a selection bias [29]. PCT is often used as a marker of systemic infection, but its role in the diagnosis of local infections (such as PJI) is limited because the threshold of PCT in patients with local infections substantially overlaps with its normal range [30]. We herein found that with regard to PJI diagnosis, in comparison with CRP, there was no significant difference in the overall test results of IL-6 and PCT. This result is the same as that previously reported in the literature, i.e., studies have not confirmed that IL-6 and PCT are superior to traditional blood biomarkers for diagnosing PJI [31].

As with other methods to diagnose PJI, NGS also has both advantages and disadvantages. The main advantage of NGS is unbiased sampling, leading to the identification of known as well as novel organisms [32]. Further, NGS can provide auxiliary genomic information needed to predict drug resistance [33]. It can also generate quantitative or semiquantitative data pertaining to bioconcentration by counting sequencing reads, which is very useful for samples containing multiple microorganisms or in case of disease processes involving more than one pathogen [34]. The main inherent disadvantage of NGS is that microbial nucleic acids in a sample can be affected by the presence of human nucleic acids. The vast majority of sequences (usually > 99%) come from human hosts, consequently limiting the overall analytical sensitivity of pathogen detection methods [35]. Another potential challenge is that testing samples, reagents, or contaminating microorganisms in the laboratory environment can affect the accuracy of results [36, 37]. In addition, while metagenes can be used to detect pathogenic bacteria, they cannot be used for drug sensitivity tests at the same time, which remains a persistent issue. The third disadvantage is that NGS is not a feasible approach for routine testing, because the price is still high at $500 or more per test.

This study had some limitations: (1) The sample size was relatively small. (2) Choosing the MSIS criteria as the gold standard for diagnosing PJI may cause grouping errors. This can explain the results that bacteria were detected in the samples of two separate patients in non-infection group. (3) Benefit analysis was not performed for NGS.

Our results nevertheless clearly indicated that NGS had higher accuracy and sensitivity than the bacterial culture method and commonly used serological biomarkers, and thus, we report that its diagnostic value to detect PJI is relatively much higher.

## Data Availability

The datasets supporting the conclusions of this article are not available in an open access repository because the authors have not finished the data analysis yet. If anyone is interested in exploring specific issue, please contact Prof. Dawei Wang.

## Abbreviations

NGS: Next-generation sequencing
PJI: Periprosthetic joint infection
ESR: Erythrocyte sedimentation rate
CRP: C-reactive protein
PCT: Procalcitonin
IL-6: Interleukin-6
AUC: Area under the curve
ROC: Receiver operating characteristic curve

## References

[1] Delanois RE, Mistry JB, Gwam CU, Mohamed NS, Choksi US, Mont MA (2017). Current epidemiology of revision Total knee Arthroplasty in the United States. J Arthroplast.

[2] Gwam CU, Mistry JB, Mohamed NS, Thomas M, Bigart KC, Mont MA (2017). Current epidemiology of revision Total hip Arthroplasty in the United States: National Inpatient Sample 2009 to 2013. J Arthroplast.

[3] Goswami K, Parvizi J, Maxwell CP (2018). Current recommendations for the diagnosis of acute and chronic PJI for hip and knee-cell counts, alpha-Defensin, leukocyte esterase, Next-generation Sequencing. Curr Rev Musculoskelet Med.

[4] Zmistowski B, Karam JA, Durinka JB, Casper DS, Parvizi J (2013). Periprosthetic joint infection increases the risk of one-year mortality. J Bone Joint Surg Am.

[5] Tarabichi M, Shohat N, Goswami K, Alvand A, Silibovsky R, Belden K (2018). Diagnosis of Periprosthetic joint infection: the potential of next-generation sequencing. J Bone Joint Surg Am.

[6] Unter Ecker N, Suero EM, Gehrke T, Haasper C, Zahar A, Lausmann C (2019). Serum C-reactive protein relationship in high- versus low-virulence pathogens in the diagnosis of periprosthetic joint infection. J Med Microbiol.

[7] Bonanzinga T, Zahar A, Dütsch M, Lausmann C, Kendoff D, Gehrke T (2017). How reliable is the alpha-defensin immunoassay test for diagnosing Periprosthetic joint infection? A prospective study. Clin Orthop Relat Res.

[8] Shahi A, Alvand A, Ghanem E, Restrepo C, Parvizi J (2019). The leukocyte esterase test for Periprosthetic joint infection is not affected by prior antibiotic administration. J Bone Joint Surg Am.

[9] Qu PF, Xu C, Fu J, Li R, Chai W, Chen JY (2019). Does serum interleukin-6 guide the diagnosis of persistent infection in two-stage hip revision for periprosthetic joint infection. J Orthop Surg Res.

[10] Alvand A, Rezapoor M, Parvizi J (2017). The role of biomarkers for the diagnosis of implant-related infections in Orthopaedics and trauma. Adv Exp Med Biol.

[11] Erdemli B, Özbek EA, Başarir K, Karahan ZC, Öcal D, Biriken D (2018). Proinflammatory biomarkers’ level and functional genetic polymorphisms in periprosthetic joint infection. Acta Orthop Traumatol Turc.

[12] Lee YS, Koo KH, Kim HJ, Tian S, Kim TY, Maltenfort MG (2017). Synovial fluid biomarkers for the diagnosis of Periprosthetic joint infection: a systematic review and meta-analysis. J Bone Joint Surg Am.

[13] Omar M, Petri M, Hawi N, Krettek C, Eberhard J, Liodakis E (2018). Higher sensitivity of swab polymerase chain reaction compared with tissue cultures for diagnosing periprosthetic joint infection. J Orthop Surg (Hong Kong).

[14] Jun Y, Jianghua L (2018). Diagnosis of Periprosthetic Joint Infection Using Polymerase Chain Reaction: An Updated Systematic Review and Meta-Analysis. Surg Infect.

[15] Salzberg SL, Breitwieser FP, Kumar A, Hao H, Burger P, Rodriguez FJ (2016). Next-generation sequencing in neuropathologic diagnosis of infections of the nervous system. Neurol Neuroimmunol Neuroinflamm.

[16] Wilson MR, Naccache SN, Samayoa E, Biagtan M, Bashir H, Yu G (2014). Actionable diagnosis of neuroleptospirosis by next-generation sequencing. N Engl J Med.

[17] Tarabichi M, Shohat N, Goswami K, Parvizi J (2018). Can next generation sequencing play a role in detecting pathogens in synovial fluid. Bone Joint J.

[18] Parvizi J, Zmistowski B, Berbari EF, Bauer TW, Springer BD, Della Valle CJ (2011). New definition for periprosthetic joint infection: from the workgroup of the musculoskeletal infection society. Clin Orthop Relat Res.

[19] Street TL, Sanderson ND, Atkins BL, Brent AJ, Cole K, Foster D (2017). Molecular diagnosis of orthopedic-device-related infection directly from sonication fluid by metagenomic sequencing. J Clin Microbiol.

[20] Margulies M, Egholm M, Altman WE, Attiya S, Bader JS, Bemben LA (2005). Genome sequencing in microfabricated high-density picolitre reactors. Nature..

[21] Yoon HK, Cho SH, Lee DY, Kang BH, Lee SH, Moon DG (2017). A review of the literature on culture-negative Periprosthetic joint infection: epidemiology, diagnosis and treatment. Knee Surg Relat Res.

[22] Palan J, Nolan C, Sarantos K, Westerman R, King R, Foguet P (2019). Culture-negative periprosthetic joint infections. EFORT Open Rev.

[23] Berbari EF, Marculescu C, Sia I, Lahr BD, Hanssen AD, Steckelberg JM (2007). Culture-negative prosthetic joint infection. Clin Infect Dis.

[24] Parikh MS, Antony S (2016). A comprehensive review of the diagnosis and management of prosthetic joint infections in the absence of positive cultures. J Infect Public Health.

[25] Million M, Bellevegue L, Labussiere AS, Dekel M, Ferry T, Deroche P (2014). Culture-negative prosthetic joint arthritis related to Coxiella burnetii. Am J Med.

[26] Lu G, Li T, Ye H, Liu S, Zhang P, Wang W (2020). D-dimer in the diagnosis of periprosthetic joint infection: a systematic review and meta-analysis. J Orthop Surg Res.

[27] Bingham JS, Hassebrock JD, Christensen AL, Beauchamp CP, Clarke HD, Spangehl MJ (2019). Screening for Periprosthetic Joint Infections With ESR and CRP: The Ideal Cutoffs. J Arthroplast.

[28] Yoon JR, Yang SH, Shin YS (2018). Diagnostic accuracy of interleukin-6 and procalcitonin in patients with periprosthetic joint infection: a systematic review and meta-analysis. Int Orthop.

[29] Di Cesare PE, Chang E, Preston CF, Liu CJ (2005). Serum interleukin-6 as a marker of periprosthetic infection following total hip and knee arthroplasty. J Bone Joint Surg Am.

[30] Shaikh MM, Hermans LE, van Laar JM (2015). Is serum procalcitonin measurement a useful addition to a rheumatologist’s repertoire? A review of its diagnostic role in systemic inflammatory diseases and joint infections. Rheumatology (Oxford).

[31] Glehr M, Friesenbichler J, Hofmann G, Bernhardt GA, Zacherl M, Avian A (2013). Novel biomarkers to detect infection in revision hip and knee arthroplasties. Clin Orthop Relat Res.

[32] Gu W, Miller S, Chiu CY (2019). Clinical metagenomic next-generation sequencing for pathogen detection. Annu Rev Pathol.

[33] Talundzic E, Ravishankar S, Kelley J, Patel D, Plucinski M, Schmedes S (2018). Next-Generation Sequencing and Bioinformatics Protocol for Malaria Drug Resistance Marker Surveillance. Antimicrob Agents Chemother.

[34] Salipante SJ, Hoogestraat DR, Abbott AN, SenGupta DJ, Cummings LA, Butler-Wu SM (2014). Coinfection of Fusobacterium nucleatum and Actinomyces israelii in mastoiditis diagnosed by next-generation DNA sequencing. J Clin Microbiol.

[35] Thoendel M, Jeraldo PR, Greenwood-Quaintance KE, Yao JZ, Chia N, Hanssen AD (2016). Comparison of microbial DNA enrichment tools for metagenomic whole genome sequencing. J Microbiol Methods.

[36] Wilson MR, O'Donovan BD, Gelfand JM, Sample HA, Chow FC, Betjemann JP (2018). Chronic meningitis investigated via metagenomic next-generation sequencing. JAMA Neurol.

[37] Bukowska-Ośko I, Perlejewski K, Nakamura S, Motooka D, Stokowy T, Kosińska J, et al. Sensitivity of Next-Generation Sequencing Metagenomic Analysis for Detection of RNA and DNA Viruses in Cerebrospinal Fluid: The Confounding Effect of Background Contamination. Adv Exp Med Biol. 2016. 10.1007/5584_2016_42. 10.1007/5584_2016_42 27405447

